# Customized In Situ Functionalization of Nanodiamonds with Nanoparticles for Composite Carbon-Paste Electrodes

**DOI:** 10.3390/nano10061179

**Published:** 2020-06-17

**Authors:** Raquel Montes, Gerard Sánchez, Jingjing Zhao, Cristina Palet, Mireia Baeza, Julio Bastos-Arrieta

**Affiliations:** 1 GENOCOV Research Group, Department of Chemical, Biological and Environmental Engineering, School of Engineering, Universitat Autònoma de Barcelona, Carrer de les Sitges, 08193 Bellaterra (Cerdanyola del Vallès), Spain; raquel.montes@uab.cat; 2 GENOCOV Research Group, Department of Chemistry, Faculty of Science, Edifici C-Nord, Universitat Autònoma de Barcelona, Carrer dels Til·lers, 08193 Bellaterra (Cerdanyola del Vallès), Spain; gerarg_93@hotmail.com; 3 Grup de Tècniques de Separació en Química, Department of Chemistry, Facultat de Ciències, Universitat Autònoma de Barcelona, Carrer dels Til·lers, 08193 Bellaterra (Cerdanyola del Vallès), Spain; zhaojingjing186@sina.com (J.Z.); cristina.palet@uab.cat (C.P.); 4 Physical Chemistry TU Dresden, Zellescher Weg 19, 01069 Dresden, Germany

**Keywords:** carbon nanostructures, nanodiamonds, graphite, optimal composition, metal nanoparticles, electrochemical (bio)sensor, surface functionalization

## Abstract

The incorporation of nanomaterials on (bio)sensors based on composite materials has led to important advances in the analytical chemistry field due to the extraordinary properties that these materials offer. Nanodiamonds (NDs) are a novel type of material that has raised much attention, as they have the possibility of being produced on a large scale by relatively inexpensive synthetic methodologies. Moreover, NDs can present some other interesting features, such as fluorescence, due to surface functionalization and proved biocompatibility, which makes them suitable for biomedical applications. In addition, NDs can be customized with metallic nanoparticles (NPs), such as silver or gold, in order to combine the features of both. Raw NDs were used as modifiers of sensors due to the electrocatalytic effect of the sp^2^ and oxygenated species present on their surface. The aim of this research work is evaluating the applicability of NDs modified with silver (Ag@NDs) and gold (Au@NDs) nanoparticles for the development of a suitable (bio)sensing platform. A complete morphological and electrochemical characterization as a function of the prepared nanocomposite composition was performed in order to improve the electroanalytical properties of the developed (bio)sensors. In the present work, the optimal composition for Au@NDs present on the nanocomposite matrix is 3.5% and the one for Ag@NDs is 1%. Good results were obtained in the evaluation of the optimal composition towards hydrogen peroxide and glucose as a model analyte using a (bio)sensor based on graphite-epoxy-Ag@NDs (17:82:1).

## 1. Introduction

Carbon-based nanocomposite materials used for (bio)sensing platforms, include different carbon nanallotropic forms (e.g., graphite, graphene) that led to electroanalytical improvements [1,2] in comparison to conventional solid electrodes, such as robustness, renewable surface, or a small background current. Composite electrodes can be customized with a variety of nanomaterials including electrocatalysts, enzymes, metal nanoparticles (NPs), quantum dots, proteins, ligands, and other chemical (bio)recognition agents [3,4]. In addition, this modification can be either within the matrix itself or on the surface.

Nanodiamonds (NDs) are another carbon nanoallotrope that recently have been widely introduced as components of (bio)sensing platforms. This is due to their cost effective large scale production with narrow size distribution, biocompatibility, and their surface chemistry, that make them suitable for further customization with different functional moieties [5]. Even though bulk diamonds are well known isolators, features attributed to the nanoscale make NDs to be electrochemically active [6,7]. This can be explained because of the existence of discrete electronic states within the NDs band gap (overlapping of surface sp^2^ orbitals), which provides NDs with a surface suitable to undergo redox reactions, and consequently have an electrochemical response [8,9].

All these characteristics make it interesting to evaluate the effect of the customization of NDs to carbon nanocomposite (bio)sensors by three different routes [10]:

(a) Covalent or noncovalent functionalization of the NDs with the modifiers and their subsequent mixing within the polymer.
(b) Directly introduce the modifiers in the carbon/polymer matrix during the preparation, which remain embedded or trapped in the nanocomposite.
(c) Immobilization of the (bio)species on the surface of the already prepared nanocomposite electrode [11].

The final properties of the nanocomposite electrodes depend on their composition, which influence highly on their electroanalytical response [12]. Up to now the principle applied to the optimization of the composite proportions has been done using the percolation theory, under the criteria of maximizing the conductive particle loading, without losing its physical and mechanical stability. The nanocomposite becomes conductive above a critical value, which is called the percolation threshold and defines the insulator–conductor transition. At this point, the first conductive network is formed through the matrix. In order to reach the electrical percolation threshold and therefore be electrically conductive, a direct physical connection or overlapping of the conductive filler is not necessary [13]. Recently, complementary strategies of characterization have been established, which demonstrate that if the composite proportions are optimized, the response of the electrode will be improved [11,13].

Electrochemical impedance spectroscopy (EIS) is a powerful technique, which offers information about the electron-transfer rate (*R_ct_*), the double-layer capacitance (*C_dl_*), contact resistance and resistance of the solution (*R_Ω_*) [14]. The electroanalytical properties required by an electrode in order to ensure enough sensitivity, a high signal-to-noise ratio, and low detection limit are: High electron-transfer rate, the lowest ohmic resistance, and double-layer capacitance. Using the EIS technique, it is possible to determine the optimal composite composition that exhibits these electrochemical properties associated with the improvement of the electroanalytical performance. Complementary techniques such as cyclic voltammetry (CV) allows obtaining supplementary information obtained by the EIS [15].

Montes et al. (2015) studied the optimization of the conductive particle distribution and the amount of the biological material inside the nanocomposite electrodes. The authors showed how these factors led to an improvement of electrochemical properties such as signal stability and limit of detection. In this case, for biocomposites based on graphite-epoxy and modified with glucose oxidase (GOD), as an enzyme model [16]. In addition, this work demonstrated that optimized electrodes with 17% of graphite and 1% of biological material presented good sensitivity and wide linear range towards glucose, as well as the limit of detection achieved was lower compared to the nonoptimized biocomposites. Later, Montes et al. (2016) reported that optimized immunosensors based on 17% of graphite and epoxy and 0.9% of RIgG on the matrix allowed obtaining the lower limit of detection using a competitive assay [17].

X-ray photoelectron spectroscopy (XPS) studies suggest that NDs are mainly composed of nonconductive sp^3^ carbon (resulting in low electroconductivity). Despite this, incorporating NDs to sensing platforms produces an electrocatalytic effect regarding changes in the mass transport. This variation can be explained because NDs block the underlying electroactive graphite matrix, creating a random array of graphite microelectrodes [9]. These facts encourage analyzing the combined effect of incorporating NDs functionalized with conductive metal NPs.

Consequently, 17% of graphite was fixed as a conductive material and the effect of the different proportions of NDs functionalized with silver and gold nanoparticles has been evaluated. Moreover, these studies concluded that to obtain a low limit of detection the amount of graphite loading it is more critical than the biological material loading in the biocomposites.

We report here the construction and characterization of nanocomposites based on graphite-epoxy and modified nanodiamonds functionalized with metallic NPs (Ag@NDs and Au@NDs). The electrochemical properties of such electrodes were studied in order to optimize the electrochemical behavior. The electroanalytical response of the optimized nanocomposites was evaluated using one reference analyte such as hydrogen peroxide. Finally, as a proof of concept of a suitable (bio)sensing platform, the optimized nanocomposite electrode was modified with glucose oxidase and applied to the detection of its enzymatic substrate.

## 2. Materials and Methods

### 2.1. Apparatus

EIS and voltammetric measurements (CV) were performed using a computer controlled Autolab PGSTAT12 potentiostat/galvanostat (EcoChemie, Utrech, The Netherlands) with a three-electrode configuration. A platinum-based electrode 53-671 (Crison Instruments, Alella, Barcelona, Spain), an AgCl covered silver wire, and the constructed graphite nanocomposite electrodes were used as a counter, reference, and working electrodes, respectively.

Linear-sweep voltagrams were performed using a computer controlled Autolab PGSTAT12 potentiostat/galvanostat (EcoChemie, Utrech, The Netherlands) with a three-electrode configuration. A single junction reference electrode Ag/AgCl Orion 900,100 (Thermo Electron Corporation, Beverly, MA, USA) with 3.0 M KCl as internal reference solution and platinum-based electrode were used as reference and auxiliary, respectively, and graphite nanocomposite electrodes as working electrodes.

Amperometric measurements were done using an amperemeter LC-4C (Bioanalytical Systems Inc., West Lafayette, IN, USA). Electroanalytical experiments were carried out using a three-electrode configuration. A single junction reference electrode Ag/AgCl Orion 900,100 (Thermo Electron Corporation, Beverly, MA, USA) with 3.0 M KCl as internal reference solution and platinum-based electrode were used as reference and auxiliary, respectively. The graphite nanocomposites electrodes were used as a working electrode. A magnetic stirrer at constant rpm provided the convective transport during the amperometric measurements.

Transmission electron microscopy (TEM) images were obtained by the high-resolution transmission electron microscopy (HR-TEM) technique using the JEOL JEM-1400 unit (Tokyo, Japan) with an acceleration voltage of 120 kV. At the same time, the energy dispersive spectroscopy (EDS) analysis was used for the qualitative determination of NPs presence.

Scanning electron microscopy (SEM) images were obtained with the Zeiss EVO^®^ MA 10 unit (Jenna, Germany) with an acceleration voltage of 15 kV.

### 2.2. Chemical Reagents

Graphite powder (average particle size of 50 μm) was received from Merck Millipore (Darmstadt, Germany) and the epoxy resin EpoTek H77 with the corresponding hardener was supplied by Epoxy Technologies (Billerica, MA, USA). Nanodiamonds (>87%) were obtained from PlasmaChem (Berlin, Germany). Potassium chloride (99.5%), potassium ferrocyanide trihydrate (>99%), potassium ferricyanide (III) (99%), nitric acid (99.5%), potassium nitrate (99%), potassium hydrogenphosphate (99.5%), potassium dihidrogenpohsphate (99.5%), silver nitrate (≥99%), gold chloride trihydrate (≥99.9%), sodium chloride (99.5%), sodium borohydride, chloroauric acid (≤99.9%), hydrogen peroxide (30%), glucose oxidase VII from *Aspergillus niger* (174,400 units/g), D-(+)-Glucose (≥99.5%), and bovine serum albumin, all of them were supplied by Sigma-Aldrich (St. Louis, MO, USA). Phosphate buffers were prepared from the potassium hydrogenphosphate (K_2_HPO_4_) and dihydrogenphosphate (KH_2_PO_4_) salts in Milli-Q water (Millipore, Billerica, MA, USA). All the dissolutions were prepared using deionized water from the Milli-Q system (Millipore, Billerica, MA, USA) with a resistivity value of 18.2 MΩ·cm.

### 2.3. Synthesis of Ag@NDs and Au@NDs

Functionalization of NDs with silver (Ag@NDs) and gold nanoparticles (Au@NDs) was carried out following an Intermatrix Synthesis approach reported in previous works [18,19], which is based on ion exchange and reduction stages to obtain a favorable distribution of nanoparticles. The NDs surface was activated with carboxylic groups by dispersing them in a 2.5 M nitric acid and placing them all in an ultrasound bath for 2 h. These groups were converted to the Na^+^ form by treating them with a 1.0 M NaCl solution with mechanical stirring for 2 h (see Equation (1)).

[NDs-COO^−^H^+^] + Na^+^ → [NDs-COO^−^Na^+^] + H^+^ (1)

Firstly, the Ag-NPs precursor (Ag^+^) is fixed on the NDs by an ion exchange stage with AgNO_3_, following Equation (2):

[NDs-COO^−^Na^+^] + Ag^+^ → [NDs-COO^−^Ag^+^] + Na^+^ (2)

Then, the appearance of the Ag^0^-NPs is achieved after the addition of NaBH_4_ (as the reducing agent). This reaction, leads the NDs to their Na^+^ form again, making suitable further synthesis of Ag^0^-NPs by this approach (as can be seen in Equation (3)).

[NDs-COO^−^Ag^+^] + NaBH_4_ → [NDs-COO^−^Na^+^] + 7/2 H_2_ + B(OH)_3_ + Ag^0^-NPs (3)

For the preparation of Au@NDs, a galvanic replacement strategy is needed, as the commonly used precursor of the Au^0^-NPs is negatively charged (AuCl_4_^−^) and therefore, its fixation on the carboxylic moieties is not possible. Thus, the difference of redox potentials between Au and Ag can be exploited, leading to a highly spontaneous galvanic replacement process to obtain Au^0^-NPs using the previously prepared Ag^0^-NPs as sacrificial templates (Equation (4)) [20].

Ag^0^-NPs + AuCl_4_^−^ → Au^0^-NPs + 4Cl^−^ + 3Ag^+^ (4)

### 2.4. Fabrication of the Electrodes

The preparation of the nanocomposite material was handmade by mixing the polymer Epotek H77A and its corresponding H77B hardener in a 20:3 (*w*/*w*) ratio and adding the conducting filler nanomaterial, graphite. The composition was fixed at 17% of graphite loading as an optimal composition for the development of amperometric nanocomposites based on previous studies [16,17]. The composite was homogenized for 30 min. After the homogenization time, the nanomaterial was incorporated, either raw NDs (1% (*w*/*w*)), NDs functionalized with gold nanoparticles (1%, 2%, and 3.5% (*w*/*w*)), or NDs functionalized with silver nanoparticles (1%, 2%, and 3% (*w*/*w*)). The composite paste was homogenized for an additional 15 min to assure the integration of the nanomaterial. The final nanocomposite paste electrode was allowed to harden during 24 h at 60 °C. Finally, the electrode surface was polished with different sandpapers of decreasing grain size. The final electrode dimensions were 28 mm^2^ and 3 mm for its geometric area and thickness, respectively. In Table 1, the different compositions evaluated on the present work are summarized.

### 2.5. Electrochemical Procedure

EIS and CV measurements were carried out in a 0.1 M potassium chloride solution containing 0.01 M potassium ferricyanide/ferrocyanide under quiescent conditions. The optimal polarization potential of the studied analytes for each prepared nanocomposite electrode was obtained by linear scan voltammetry. This technique consists of registering the intensity while a potential sweep is done in a determined direction at a constant scan rate. The sweeps are carried out without force. For each of the studied analyte a sweep potential between 0 and 1.5 V was applied at a scan rate of 10 mV·s^−1^. Firstly, a potential sweep is done on a 20 mL solution containing the support electrolyte, PBS at pH 7.0, and then successive sweeps are carried out on the same electrolytic solution after adding, in a consecutive way, different microvolumes of a solution containing the analyte of interest. For hydrogen peroxide measurements, consecutive microvolumes of a solution of 1 M hydrogen peroxide solution were added until a final concentration of 27 mM H_2_O_2_ in the electrolytic media. For glucose measurements, consecutive microvolumes of a 1 M glucose solution were added until a final concentration of 100 mM glucose in the electrolytic media.

Amperometric detection of the different reference analytes (hydrogen peroxide and glucose) was made under force convection by stirring the electrolyte solution with a magnetic stirrer. The limit of detection (LOD) was estimated by the *S*/*N* = 3 criterion [21]. The LOD was calculated three times (*n* = 3) and presented with their respective standard deviation.

### 2.6. Surface Modification of the Nanocomposite Electrodes with Glucose Oxidase

For the surface modification of the electrodes with glucose oxidase by the direct contact method, a stock solution is prepared from 15 mg of GOD and 300 μL of the bovine serum albumin (BSA) solution at 1% (*p*/*v*) in PBS. The electrode was incubated with the GOD solution using a thermomixer (Thermomixer comfort, Eppendorf AG, Hamburg, Germany) at 600 rpm and 20 °C. Three different contact times were evaluated: 30 min, 1 h and 24 h.

## 3. Results

### 3.1. Characterization of the Modified NDs Containing Composites

**Figure 1:**
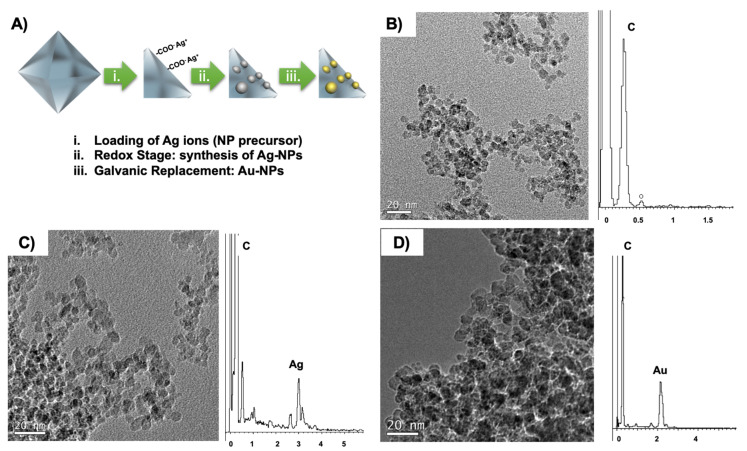
Scheme of the functionalization process (**A**), nanodiamonds (NDs) TEM image and EDS (**B**), silver nanodiamonds (Ag@NDs) TEM image and EDS (**C**), and gold nanodiamonds (Au@NDs) TEM image and EDS (**D**).

The characterization of the NDs before and after the process of modification with the metal NPs was screened by HR-TEM. Figure 1 shows the schematic representation of the NDs modification, together with the TEM images and the corresponding energy-dispersive X-ray spectroscopy (EDS) for all systems, NDs, Ag@NDs, and Au@NDs. A good monodispersity in the NDs size can be observed. Moreover, the average diameter of the NDs was <4 nm (3.5 ± 0.3), and when adding the silver and gold nanoparticles it increased up to 4.5 ± 0.3 nm for the Ag@NDs and 6.7 ± 0.5 nm for the Au@NDs. Furthermore, the EDS carried out for both Ag@NDs and Au@NDs systems confirmed the presence of the silver and gold NPs attached to the NDs.

### 3.2. Morphological Characterization by SEM

The SEM images allowed observing qualitatively the roughness and the porosity of the nanocomposites surface constructed. Figure S1 shows SEM images obtained for each ND containing nanocomposite, in comparison to the bare electrode based on graphite-epoxy (Figure S1A) at different magnification levels. It can be seen that the incorporation of the different nanomaterials to the matrix at different amounts of gold and silver NPs: NDs (Figure S1B), Au@NDs (Figure S1C–E), and Ag@NDs (Figure S1F–H) does not modify the morphology and that in all cases, the surface presents a high homogeneity and certain roughness. This homogeneity assures better reproducibility during the analytical application.

### 3.3. Impedimetric Characterization

By means of the EIS technique, the electrochemical parameters of the different nanocomposite electrodes developed have been evaluated. The obtained values for *R_Ω_*, *R_ct_*, and *C_dl_* allow predicting the electrochemical behavior as a function of the amount of nanomaterial (raw NDs, Au@NDs, or Ag@NDs) incorporated to the matrix. These properties have been compared to the ones of the bare electrode. The electroanalytical properties required by an electrode are the high electron-transfer rate (lower *R_ct_*) and the lowest resistance of the solution (*R_Ω_*) together with the lowest double-layer capacitance (*C_dl_*), in order to ensure high sensitivity and high signal-to-noise ratio and, therefore, low detection limits. The results (Figure S2A,B, Supplementary Materials) were obtained by fitting the impedance spectra to an equivalent Randles circuit (see Figure S2C, Supplementary Materials). This circuit was sufficiently suitable in order to adjust and obtain the values of *R_Ω_*, *R_ct_*, and *C_dl_*, from the point of view of the interfacial phenomena that takes places in the electrochemical cell [22]. Results for Au@NDs or Ag@NDs are presented separately.

**Figure 2:**
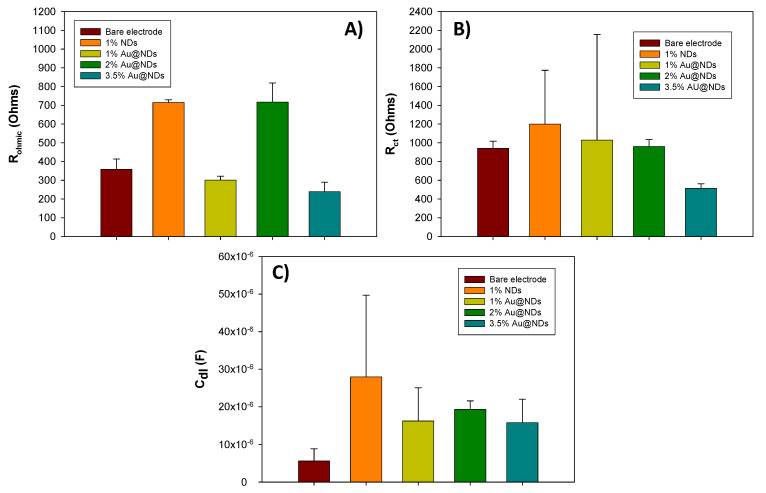
Graphs and values for the resistance of the solution (*R_Ω_*) (**A**), electron-transfer rate (*R_ct_*) (**B**), and double-layer capacitance (*C_dl_*) (**C**) of the electrodes with 17% graphite (bare electrode), 17% graphite plus 1% raw NDs, and 17% graphite plus 1%, 2%, and 3.5% Au@NDs with their corresponding standard deviation (*n* = 3). Values were extracted from the adjustment of the impedance spectrum that were obtained in a 0.1 KCl solution with an equimolar concentration of [Fe(CN)_6_]^3−^/[Fe(CN)_6_]^4−^ 0.01 M. Frequencies interval: 100 KHz–100 mHz.

Figure 2 presents the values for *R_Ω_*, *R_ct_*, and *C_dl_* and shows the comparison between the bare electrode containing 17% graphite and the modified electrodes with 17% graphite plus 1% raw NDs and 17% graphite plus 1%, 2%, and 3.5% Au@NDs. An increase in the electrochemical parameters’ values are observed when 1% of raw NDs are incorporated to the matrix. This increase is associated to the separation in the conductive particles present on the surface electrodes. When it is related to the electrodes with different amounts of Au@NDs, the electrode with 17% graphite plus 3.5% Au@NDs is the one with the lowest ohmic resistance (238.64 Ω) and charge-transfer resistance (513.54 Ω). Regarding the double-layer capacitance values, no significant difference between the three electrode compositions containing gold is observed. According to these results, the one containing 3.5% Au@NDs would be the optimal in this case due to this composition that presents the lowest ohmic resistance, charge-transfer resistance, and double-layer capacitance.

**Figure 3:**
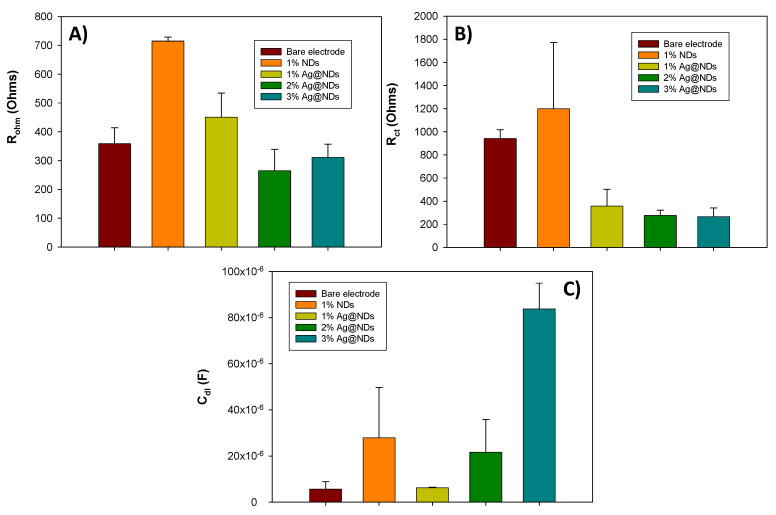
Graphs and values for the *R_Ω_* (**A**), *R_ct_* (**B**), and *C_dl_* (**C**) of electrodes with 17% graphite (bare electrode), 17% graphite plus 1% raw NDs, and 17% graphite plus 1%, 2%, and 3.5% Ag@NDs with their corresponding standard deviation (*n* = 3). Values were extracted from the adjustment of the impedance spectrum that were obtained in a 0.1 M KCl solution with an equimolar concentration of [Fe(CN)_6_]^3−^/[Fe(CN)_6_]^4−^ 0.01 M. Frequencies interval: 100 KHz–100 mHz.

Figure 3 compares the values for *R_Ω_*, *R_ct_*, and *C_dl_* of the 17% graphite electrode, 17% graphite plus 1% raw NDs electrode, and 17% graphite plus 1%, 2%, and 3% Ag@NDs electrodes. Looking at the resistances’ values, there is a decrease in the ohmic resistance and charge-transfer resistance when the amount of Ag@NDs increases. The charge-transfer resistance compared to the bare electrode is lower, so the incorporation of the Ag@NDs on the matrix produces an enhancement on the electrode sensitivity and response time. Taking into the account these values, it could be determined that the electrode with 17% graphite plus 2% Ag@NDs would be the optimal composition, but it is necessary to consider the double-layer capacitance values as well. There is an increase in this value when the amount of Ag@NDs also increases. Compared to the bare electrode (5.62 × 10^−6^ F), the electrode with 2% Ag@NDs (2.16 × 10^−5^ F) has a high value of this parameter (greater than the 1% Ag@NDs, 6.23 × 10^−6^ F) and it could lead to a high signal-to-noise ratio or even mask the faradic signal. According to these results, the 1% Ag@NDs electrode would be the optimal since there is not much difference in the resistance values (*R_Ω_* and *R_ct_*), and the double-layer capacitance value is half of the 2% Ag@NDs.

The comparison between the optimal Au@NDs proportion (3.5%) and the optimal Ag@NDs proportion (1%) was made in order to evaluate the effect of both compositions of metallic load. Looking at the *R_Ω_* values it can be seen that a slight difference between them, 238.64 Ω for the optimal with Au@NDs and 450.57 Ω for the optimal with Ag@NDs, could make the electrode with 1% of Au@NDs present better sensitivity and low response time. However, when it is compared to the *R_ct_* value, the Au@NDs electrode (513.54 Ω) is higher than the resistance of the Ag@NDs electrode (357.85 Ω). The C_dl_ value of the Au@NDs (1.57 × 10^−5^ F) electrode is twice the Ag@NDs (6.23 × 10^−6^ F) electrode’s capacitance. For this reason and taking into the account the properties required for electroanalytical purposes, such as a rapid response time, low limit of detection, and high sensitivity, the 1% Ag@NDs electrode is better than the 3.5% Au@NDs in terms of electrochemical properties.

### 3.4. Cyclic Voltammetry Measurements

**Figure 4:**
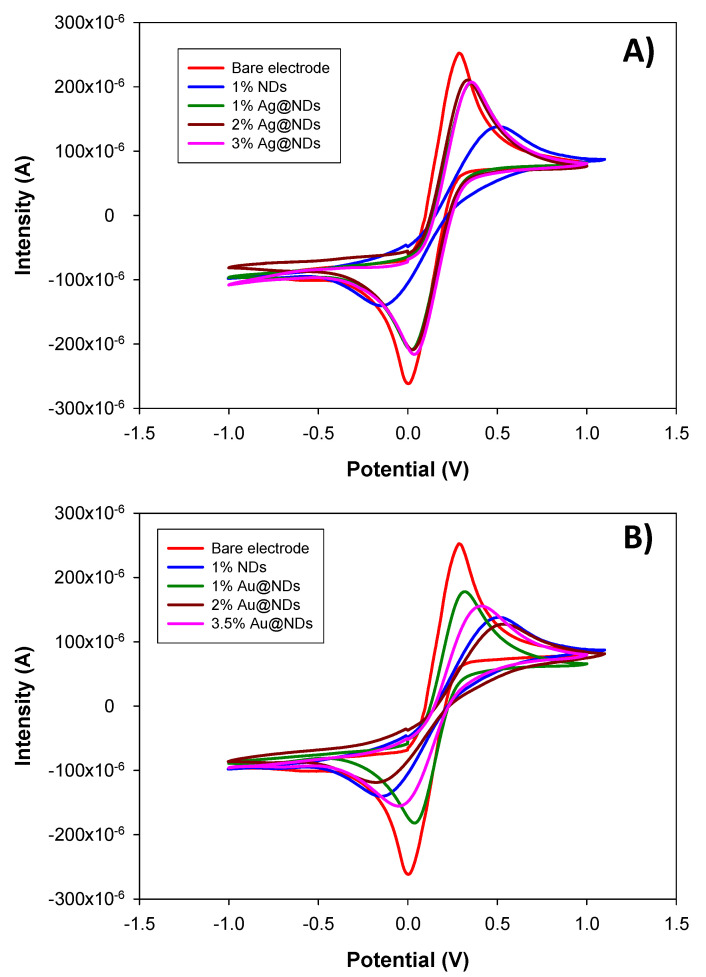
Superposition of the cyclic voltammograms (CV) obtained for the electrodes with 17% graphite, 17% graphite plus 1%, 2%, and 3% Ag@NDs (**A**). Superposition of the CV obtained for electrodes with 17% graphite, 17% graphite plus 1% raw NDs, and 17% graphite plus 1%, 2%, and 3.5% Au@NDs (**B**). Scan rate: 10 mV·s^−1^.

Cyclic voltammograms were taken for the different NDs containing nanocomposite electrodes and have been compared to the bare electrode based on graphite-epoxy (Figure 4).

In Figure 4A, we can see the comparison of the cyclic voltammograms regarding the electrodes containing raw NDs and Ag@NDs, and their comparison to the bare electrode. A slight difference between the voltamperograms can be seen. However, when comparing the 1% Ag@NDs electrode to the 1% raw NDs electrode, the oxidation-reduction peaks are closer for Ag@NDs than for raw NDs. If the oxidation peak and the reduction peak are very close to each other, it indicates a macroelectrode array behavior [23], in which the lineal diffusion is dominant. On the contrary, if both peaks are separated from one another, it indicates a microelectrode array behavior [23,24] and the radial diffusion will control the mass transport. When developing this kind of transducer, the microelectrode behavior is the interesting one since this will provide an optimal particle distribution that at the same time will assure lower noise levels because there is a less active area. In this case, the variations from one peak to the other are not very significant (small variations) so it can be concluded that all these electrodes have a microelectrode array behavior.

Figure 4B presents the comparison between the bare electrode and the electrodes modified with different proportions of Au@NDs and raw NDs, obtaining the same trend of the previous case. However, an increase in the amount of Au@NDs is causing an offset between the cathode and anodic peak. The voltamperograms present a behavior more similar to the one with the raw NDs than to the bare electrode. In general, these results indicate that the electrodes with different proportions of the developed Au@NDs and Ag@NDs present good reproducibility and similar electrochemical behavior.

From the cyclic voltamperograms, different parameters as the peak intensity, the separation potential (between the oxidation and reduction peak), and the active area of the electrode can be extracted. The active area of the electrode can be estimated from the values of the peak intensities obtained by Randles–Sevčik (Equation (5)) [22]: Where *α* corresponds to the transference coefficient (0.5), *D_red_* is the diffusion coefficient of the reduced species (6.32 × 10^−6^ cm^2^·s^−1^), *ʋ* is the scanning speed (0.01 V·s^−1^), A is the electroactive area, and C*_red_ is the bulk concentration of the electroactive species (0.01 M).

*I_p_* = 3.01 × 10^5^*n*^3/2^ (*α D_red_ ʋ*)^1/2^ A C*_red_ (5)

The values for the electroactive area of each electrode are listed in Table 2. The bare electrode containing only graphite presents an electroactive area of 0.43 ± 0.09 cm^2^ but when it is modified with 1% of NDs there is a decrease on the electroactive area (down to 0.27 ± 0.03 cm^2^) as the NDs are separating the conductive particles present on the matrix. This separation of the conductive particles is compensated by the previous functionalization of the NDs with Ag and Au nanoparticles. It is verified that when increasing the percentage of NP@NDs the electroactive area increases, in addition the Ag functionalization supposes a greater increase. This fact can be explained by the intrinsic differences in the process of functionalization by galvanic replacement [18,20]. During the process of functionalization of NDs with Au, three Ag atoms are replaced galvanically by one Au atom. Consequently, the resulting amount of Au in Au@NDs is lower than the amount of Ag in Ag@NDs and the electroactive area is lower. When the matrix is modified with different proportions of Ag@NDs, there is a clear upward trend: The more Ag@NDs percentage the higher the electroactive area the electrode presents. This is due to the incorporation of conducting particles that are introduced into the matrix, although this fact modifies the spatial separation and inner distribution of the conductive particles.

For electrodes with Au@NDs, it seems that higher percentages of Au@NDs are needed to increase the electroactive area with respect to the 1% raw NDs electrode. To compensate this problem, a higher percentage of the conducting material (Au NPs) may be necessary, to obtain an electroactive area that is similar to the case of 1% raw NDs.

Taking into the account the results obtained by EIS and CV techniques, it is suggested that the nanocomposite modified with 1% of Ag@NDs seems to be the optimal proportion in terms of the electrochemical response features.

### 3.5. Electroanalytical Evaluation

Once the best composition regarding the electrochemical properties for the electrodes based on graphite-epoxy and Ag@NDs was determined (17% graphite + 1% Ag@NDs), the electroanalytical properties of the electrode were evaluated and compared to the ones for the bare electrode. Firstly, the characterization was done by the determination of hydrogen peroxide as a model analyte.

#### 3.5.1. Evaluation of the Working Potential

To study the catalytic effect of introducing small amounts of Ag@NDs in the electrode’s matrix, firstly the polarization potential for hydrogen peroxide has to be determined. To do so, a study using the linear sweep voltammetry (LSV) technique was carried out at different concentrations of analyte and in the absence of analyte for two of the electrodes: The one containing 1% Ag@NDs, which was found to present optimal electrochemical properties, and the one without any modifier (bare electrode).

Figure S3 (see Supplementary Materials) shows both lineal voltamperograms for the electrode containing only the graphite-epoxy (17% graphite) and the Ag@NDs containing electrode (17% graphite + 1% Ag@NDs). The oxidation of the hydrogen peroxide using the bare electrode is obtained at 900 mV. However, for the electrode modified with Ag@NDs the potential is reduced to 750 mV. Therefore, the introduction of the Ag@NDs in the nanocomposite matrix enhances the electrochemical detection of this analyte reducing the working potential by 17%. This fact confers selectivity to the sensor because it decreases the number of species that can be oxidized.

#### 3.5.2. Electroanalytical Parameters of Response

The electroanalytical parameters of response such as sensitivity and limit of detection have been compared for both sensors (bare and 1% Ag@NDs electrode). Measurements were carried out using the hydrodynamic amperometry technique at 750 mV as the working potential. The LOD measurements were performed by triplicate and experimental errors are expressed as the standard deviation. By adding 1% Ag@NDs to the composite transducers, no significant differences have been observed regarding the sensitivity, 0.01 µA·L·mg^−1^ for bare electrode and 0.09 µA·L·mg^−1^ for 1% Ag@NDs. A similar trend is observed regarding the limit of detection for hydrogen peroxide, 0.068 ± 0.001 and 0.095 ± 0.003 mg·L^−1^ for bare electrode and 1% Ag@NDs, respectively. However, what this modified electrode offers is the possibility of working at lower potentials and thus reducing the interferences due to the presence of other analytes that can be oxidized at the same potential, as mentioned previously. The reduction of the working potential is a considerable improvement since one of the main problems of amperometric (bio)sensors is the fact that high working potentials are needed unless a redox mediator is used. The introduction of this kind of nanoparticle could be an alternative to the redox mediator. In addition, this lessening of the polarization potential reduces the possible interferences since at a lower potential less species will be oxidized.

### 3.6. Evaluation of (Bio)sensing Approach: Analytical Response to Glucose

Optimized nanocomposites electrodes with 1% Ag@NDs, have been modified with glucose oxidase as an enzyme model in order to evaluate the analytical response to glucose. Firstly, the working potential for glucose detection after the incorporation of the enzyme by LSV has been evaluated (see Figure S4, Supplementary Materials). Figure S5 (Supplementary Materials) summarizes the analytical procedure and the reaction that takes place on the electrode’s surface. The complete reaction consists of two separate reactions: The first one is the biocatalyst reaction between the glucose oxidase and the glucose by hydrogen peroxidase production, and the second is the electroanalytical reaction when the product is oxidized on the surface electrode. This study was carried out in the presence of glucose after the 1% Ag@NDs electrode was incubated with glucose oxidase for 30 min. The results showed that the oxidation potential is 820 mV. Compared to the working potential for enzymatic (bio)sensors for glucose determination on the literature, the potential is improved by the incorporation of the nanomaterials from 1050 [16] to 820 mV, so it was reduced significantly. Consequently, the selectivity when the enzyme is placed on the surface of the Ag@NDs-tuned electrode, with respect to matrix modification was increased.

**Figure 5:**
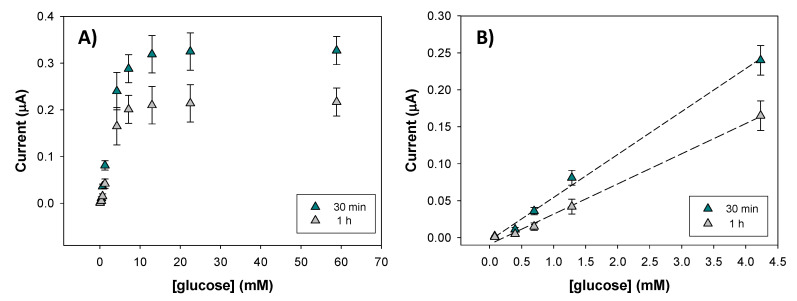
Response of the 17% graphite + 1% Ag@NDs electrodes after being mixed with glucose oxidase in the thermomixer for 30 min and 1 h for glucose concentration between 0–60 mM (**A**). Linear concentration range for *t* = 30 min and *t* = 1 h (**B**). The experimental error was calculated as the standard deviation for *n* = 3 measurements.

In order to optimize the experimental conditions, an evaluation of the influence of the contact time in the electroanalytical parameters of response has been done. The times studied were 30 min, 1 h and 24 h. Lower incubation times were evaluated but there was no response to glucose. Figure 5 shows the calibration curve (A) and the linear range (B) for the electrode with 17% graphite plus 1% Ag@NDs for an incubation with GOD of 30 min and 1 h. When the electrode surface was incubated for 24 h, the electrode’s response was saturated at lower concentrations (0.7 mM), so this experiment is not included in the Figure. Therefore, at this point it can be concluded that 24 h of contact time are not necessary in order to obtain good sensitivity, low detection limit, and a wide linear range.

Table 3 details the analytical parameters such as detection limit and sensitivity. It can be determined that there is no significant difference observed on sensitivity and detection limit when the time contact increases from 30 min to 1 h. However, the increase in the contact time produces a higher enzyme saturation at a lower glucose concentration (Figure 5A). According to these results, and in order to reduce the contact time, 30 min were considered as the optimal contact time.

Compared to other (bio)sensors based on nanomaterials [25], the LOD achieved are similar (0.09 vs. 0.08 mM in this work), so this highlights the potential of the developed (bio)sensor platform. The limit of detection achieved with a biocomposite electrode based on graphite-epoxy (17:82 (*w*/*w*)) and modified with 1% of GOD in the matrix [16] compared with the presented platform are similar: 0.04 and 0.08 mM, respectively. However, the sensitivity decreases one order of magnitude, from 0.214 to 0.058 µA/mM. It is important to highlight that the (bio)nanocomposite electrodes presented in this work containing a lower enzyme amount achieve an equivalent limit of detection towards glucose.

In order to evaluate the specific adsorption of glucose oxidase on the electrode surface, the bare electrode was incubated for the optimal time of 30 min with the enzyme solution. No changes on the current were observed after the glucose additions. Therefore, this means that the specific adsorption of the glucose oxidase on the surface of the electrode only takes place when there are Ag@NDs that are incorporated to the electrode matrix, as expected. The stability of the developed device was evaluated by the hydrodynamic amperometry. The short-term stability of the platform was investigated by repeating the determination of 0.4 and 4.2 mM glucose aliquots at the same sensor. Nine measurements for each concentration achieved a good repeatability with the relative standard deviation (RSD) of 0.03% and 0.04%, respectively. Afterwards, multiple calibration experiments were performed with the same nanocomposite sensor within a 24 h period in order to make an estimation of the loss of sensitivity to long-time. During a working day, the sensor was exposed to air without renewing their surface and no more functionalization process was required. Six calibrates were made and a sensitivity of 0.06 ± 0.01 µA·mM^−1^ (*n* = 6) and RSD of 3.74% was obtained. The low RSD value can be mainly attributed to the chemical stability of the enzyme over surface of the transducer.

## 4. Conclusions

In this work, nanocomposite sensors containing different proportions of Ag@NDs and Au@NDs were constructed and characterized by means of electrochemical impedance spectroscopy and cyclic voltammetry in order to optimize their analytical response in terms of high signal-to-noise ratio, rapid response, and low limit detection. In this sense, EIS and CV allowed us to optimize the nanocomposite composition regarding ohmic resistance (related to percolation theory), charge-transfer resistance (associated to heterogeneous electron-transfer, which depends on the electrochemical active surface), and double-layer capacitance (correlated to the background current and consequently to the signal/noise ratio). In the present work, the optimal composition for Au@NDs present on the nanocomposite matrix is 3.5% and the one for Ag@NDs is 1%. When comparing both optimal modifier compositions, 1% Ag@NDs on the electrode turned out to be the best one since despite presenting a higher ohmic resistance, it offered a lower charge-transfer resistance and double-layer capacitance.

The electroanalytical experiments showed that the incorporation of 1% Ag@NDs on the graphite/epoxy matrix allow reducing the working potential for the hydrogen peroxide from 900 mV using the bare electrode to 750 mV, which is an important feature in amperometric measurements in order to minimize the interferences.

Good results were obtained in the immobilization of an enzyme on the electrode’s surface by direct contact. The electroanalytical evaluation of glucose using the nanocomposite sensor tuned with GOD allowed reducing the working potential from 1050 to 820 mV achieving a good detection limit and sensitivity.

In conclusion, we developed an easy to develop, cheap, and robust electrochemical sensor based on nanocomposite material formed of 17% graphite and 1% Ag@NDs. This kind of electrode could become a suitable platform in terms of sensing, as long as the enzymatic reaction taking place on its surfaces produces hydrogen peroxide (any type of oxidase) that will be oxidized directly on the electrode’s surface. Moreover, further research could be addressed to enlarge this line of interest in terms of a different functionalization of the electrodes’ surface to widen the detection possibilities and applications.

## Figures and Tables

**Table 1:** Composition summary of fabricated nanocomposite electrodes.

% Graphite/% Epoxy (*w*/*w*)	% Modifier (*w*/*w*)	Nomenclature
17%/83%	-	Bare electrode
17%/82%	1% raw NDs	NDs electrode
17%/82%, 81%, and 79.5%	1%, 2%, and 3.5% Au@NDs	Au@NDs electrode
17%/82%, 81%, and 80%	1%, 2%, and 3% Ag@NDs	Ag@NDs electrode

**Table 2:** Values of the electroactive area for the different developed electrodes. The experimental error is calculated as the standard deviation for *n* = 3 measurements.

Electrode	Electroactive Area (cm^2^)	Electrode	Electroactive Area (cm^2^)
17% graphite	0.43 ± 0.09	17% graphite + 1% NDs	0.27 ± 0.03
17% graphite + 1% Ag@NDs	0.39 ± 0.01	17% graphite + 1% Au@NDs	0.30 ± 0.06
17% graphite + 2% Ag@NDs	0.40 ± 0.01	17% graphite + 2% Au@NDs	0.23 ± 0.01
17% graphite + 3% Ag@NDs	0.42 ± 0.03	17% graphite + 3.5% Au@NDs	0.29 ± 0.01

**Table 3:** Calibration parameters obtained with the hydrodynamic amperometry technique for the electrode 1% graphite + 1% Ag@NDs modified with GOD by the direct contact method at different contact times, using glucose as the analyte. The experimental error was calculated as the standard deviation for *n* = 3 measurements.

Time	Sensitivity (µA·mM^−^^1^)	LOD (mM)	Saturation Concentration (mM)
30 min	0.058 ± 0.002	0.08 ± 0.01	12.98
1 h	0.041 ± 0.003	0.08 ± 0.01	7.16
24 h	-	-	0.70

## Supplementary Materials

The following are available online at https://www.mdpi.com/2079-4991/10/6/1179/s1; Figure S1: SEM images of the graphite/epoxy electrodes; Figure S2: Nyquist plots and scheme of the equivalent circuit; Figure S3: Linear sweep voltamperograms using hydrogen peroxide as analyte; Figure S4: Linear sweep voltamperogram obtained for the 1% Ag@NDs electrode modified with GOD; Figure S5: Scheme of the analytical procedure.
