# Supplementary material for: Customized In Situ Functionalization of Nanodiamonds with Nanoparticles for Composite Carbon-Paste Electrodes
Source: Nanomaterials (Basel). 2020 Jun 17;10(6):1179. doi: 10.3390/nano10061179 (PMC7353388; doi:10.3390/nano10061179)
Supplement: Supplementary file 1 [file nanomaterials-10-01179-s001.pdf]

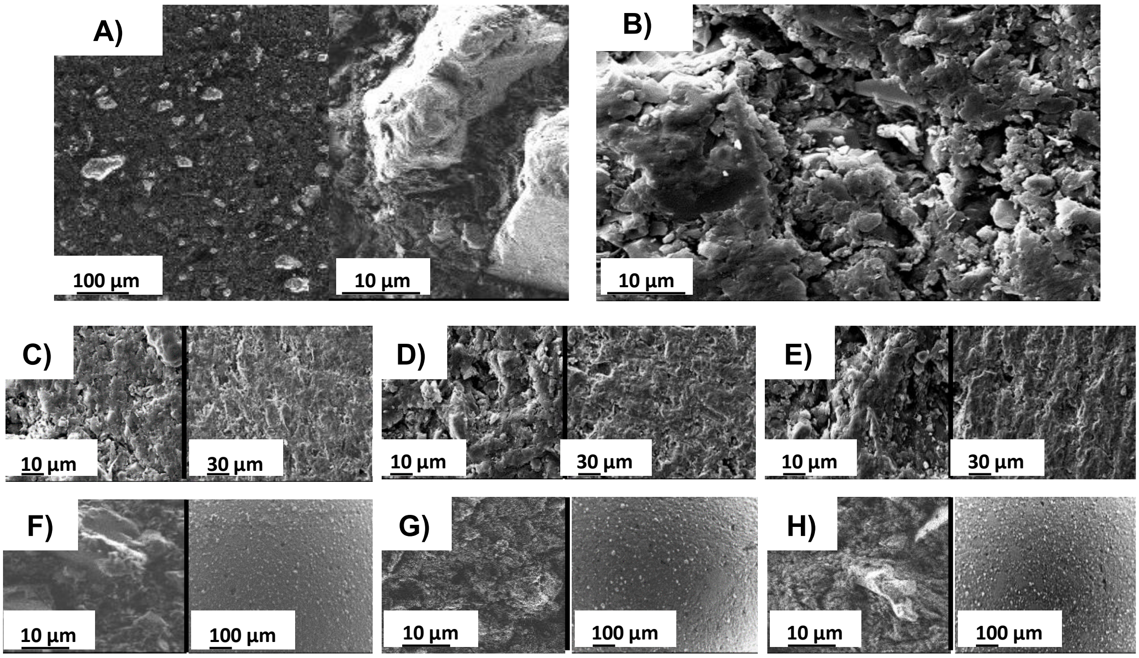

Figure S1: SEM images at different magnification levels of the graphite/epoxy electrode (**A**), the electrode containing 1% NDs (**B**), the electrodes containing 1%, 2% and 3.5% of Au@NDs (**C–E**) and the electrodes containing 1%, 2% and 3% of Ag@NDs (**F–H**).

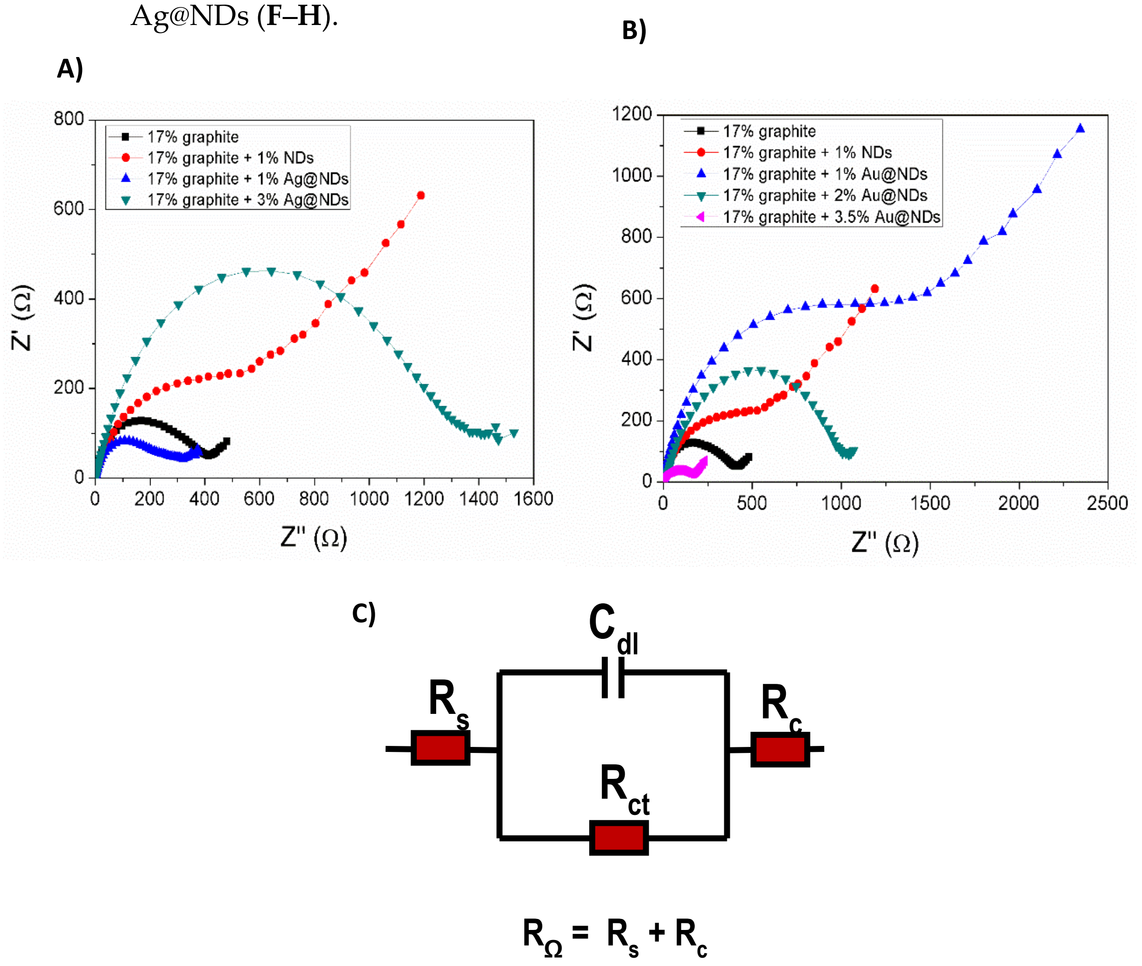

Figure S2: Comparison of Nyquist plots for bare electrode, 1% NDs, 1% Ag@NDs, 2% Ag@NDs and 3% Ag@NDs electrodes (**A**) and bare electrode, 1% NDs, 1% Au@NDs, 2% Au@NDs and 3.5% Au@NDs electrodes (**B**) in presence of 0.01 M $Fe(CN)_6^{3-}/Fe(CN)_6^{4-}$ under quiescent condition in 0.1 M KCl. Scheme of the equivalent circuit (**C**) used for the impedance spectra fitting to obtain the electrochemical parameters of ohmic resistance (R_Ω), charge-transfer resistance (R_{ct}) and double-layer capacitance (C_{dl}).

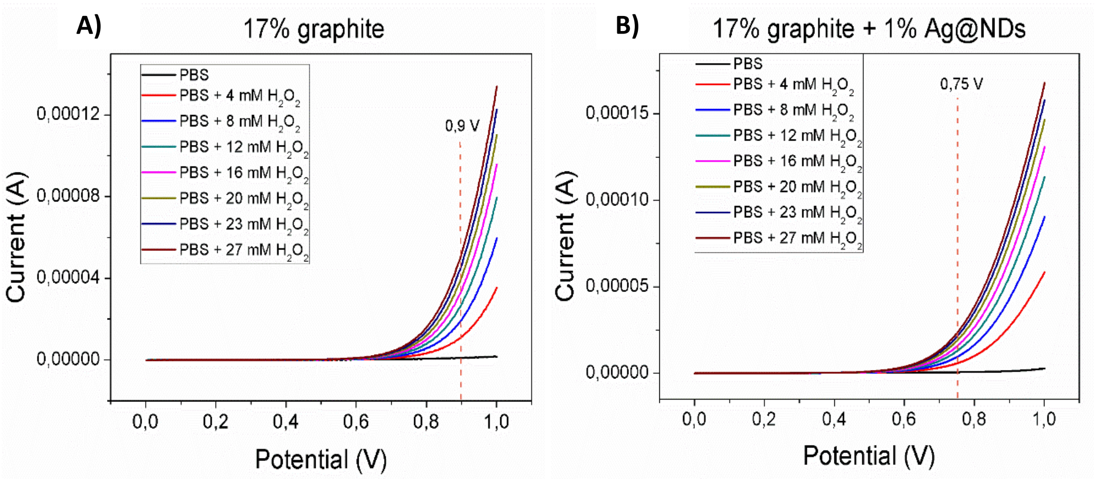

Figure S3: Linear sweep voltamperograms obtained each electrode using hydrogen peroxide as analyte in electrolitic medium KCl 0.1 M and phosphate buffer 0.1 M at pH 7.0. 17% graphite (bare electrode) (**A**) and 17% graphite + 1% Ag@NDs (**B**). The dashed lines indicate the working potential applied to each electrode.

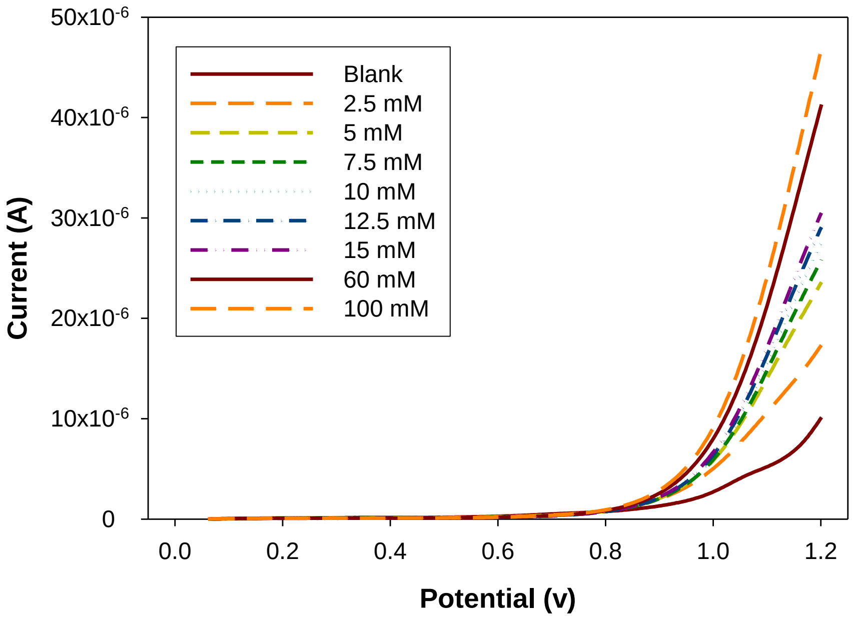

Figure S4: Linear sweep voltamperogram obtained for the 1% Ag@NDs electrode modified with GOD in presence of glucose in electrolitic medium KCl 0.1 M and phosphate buffer 0.1 M at pH = 7.0.

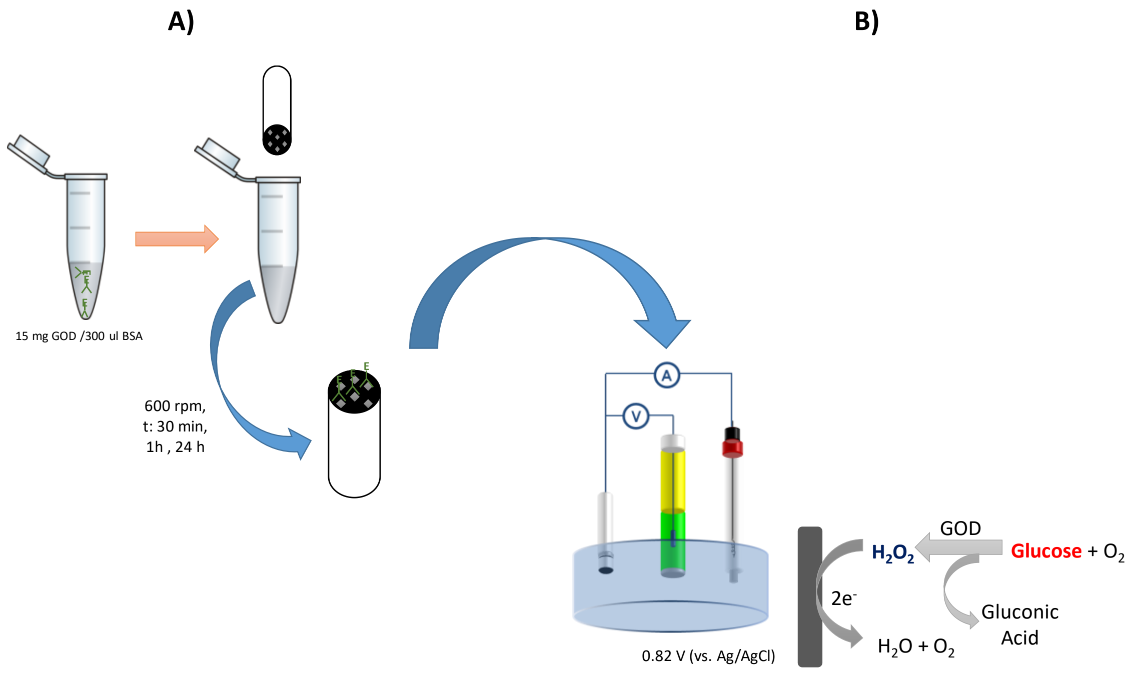

Figure S5: Scheme of the analytical procedure and the reaction that takes place on the surface electrode. Incubation step (**A**). The reaction that takes place in two stages: first one the biocatalyst reaction between the glucose oxidase and the glucose by hydrogen peroxidase production, and secondly the electroanalytical reaction when the product is oxidized on surface electrode (**B**).
